# The importance of core strength for change of direction speed

**DOI:** 10.3389/fphys.2024.1376422

**Published:** 2024-03-26

**Authors:** Henrieta Horníková, Erika Zemková

**Affiliations:** ^1^ Department of Track and Field and Sport Conditioning, Faculty of Physical Education and Sport, Comenius University Bratislava, Bratislava, Slovakia; ^2^ Department of Biological and Medical Sciences, Faculty of Physical Education and Sport, Comenius University Bratislava, Bratislava, Slovakia

**Keywords:** athletes, core muscles, core stability, core training, running speed

## Abstract

Change of direction speed (CODS) is determined by several physical aspects, such as linear sprint speed, reactive strength and power of leg muscles. It appears that core strength may also play a role in CODS, however, its relationship to CODS remains unclear. The aim of this narrative review was to analyze the literature addressing a) the relationship between core strength and CODS and b) the effect of core strength training on CODS. This analysis revealed a significant relationship between the parameters of core strength and stability (the pressure of the activated core muscles during lower limb movement and the greatest mean force output of maximum volunteered contraction) and the time in the Agility T-Test. However, this parameter was not significantly related to the strength endurance of core muscles (total time in the plank test). Core training provides a sufficient stimulus for the development of CODS in less-skilled middle-adolescent athletes, while its effectiveness decreases in higher-skilled adult athletes. These findings indicate that core muscle strength contributes significantly to the change of direction speed. Core training is therefore useful for improving CODS.

## 1 Introduction

Running is the most dominant type of locomotion in various sports such as court (tennis, squash, badminton, *etc.*) and team sports (soccer, handball, basketball, *etc.*). In this environment, there are many external stimuli (ball movements, movements of teammates and opponents, *etc.*) on the limited size of the playing field, forcing the player to change direction of running very frequently. These types of runs can be pre-planned, also known as change of direction speed, or non-planned as a response to external stimuli known as agility (Young et al., 2015). It can be assumed that these changes of direction sprints with multiple accelerations and decelerations are potentially one of the most important physical factors of athletes’ performance in these sports (Bloomfield et al., 2007; Brughelli et al., 2008). It is known that the change of direction speed is determined by factors such as straight sprinting speed, and leg muscle qualities (strength, power, and reactive strength) (Young et al., 2002).

In recent years, the importance of core strength in change of direction sprints has also been discussed (Young et al., 2015). Some researchers consider the core strength as a determinant of CODS (Young et al., 2015) because they assume that an increase in core strength leads to higher athletic performance (Imai and Kaneoka, 2014; Ingebrigtsen et al., 2014). The question arises as to how many studies have investigated this issue in the last 8 years. Apparently, they assumed that core strength refers to the muscle control of the lumbo-pelvic region to maintain functional stability and ensure optimal energy transfer from the trunk to the distal segments (Akuthota and Nadler, 2004). The results of several studies showed that core strength correlated significantly with CODS (Nesser et al., 2008; Imai and Kaneoka, 2016; de Bruin et al., 2021; Ahmed et al., 2022), however, some of them revealed only a weak or poor correlation (Imai and Kaneoka, 2016; de Bruin et al., 2021). On the other hand, Cengizhan et al. (2019) did not find a significant correlation between core endurance and CODS in football players and recreationally active men. Therefore, the question remains whether and to what extent the core strength contributes to CODS in athletes. The aim of this narrative review was to analyze the literature that deals with a) the relationship between core strength and CODS and b) the effect of core strength training on CODS.

## 2 Association between core strength and CODS

A literature search was conducted with Semantic Scholar, ResearchGate and Academia. The search strategy included a combination of these terms: “relationship” AND “core strength” AND “change of direction speed” AND “athletes”. The main inclusion criterion was that studies investigated correlations between core strength and CODS in young athletes (up to 30 years old) of different sports (cross-sectional studies only), regardless of gender and level of performance. Studies published before the year 2008 were excluded. A total of five studies investigated the relationship between core muscle strength and CODS (Nesser et al., 2008; Imai and Kaneoka, 2016; Cengizhan et al., 2019; de Bruin et al., 2021; Ahmed et al., 2022). Ahmed et al. (2022) found a significant negative correlation between the pressure of the activated core muscles during lower limb movement and time in change of direction speed test in young professional badminton players. Significant negative correlations were also revealed between total core endurance score in plank tests and time in the change of direction speed test in National Collegiate Athletic Association Division I football players, but not with right and left flexion endurance times (Nesser et al., 2018). In another study using this field-based setting, CODS was also only associated with total score in the core endurance tests but not with the side plank tests in male adolescent soccer players (Imai and Kaneoka, 2016). In the study by Cengizhan et al. (2019), no significant association was found between the CODS test and core endurance times in plank in young male professional basketball players.

A more complex study by de Bruin et al. (2021) investigated the relationship between core strength, muscular endurance and CODS in female athletes of different sports. Different results were obtained depending on the type of core strength assessment. CODS correlated significantly with the core strength measured by Biering-Sørensen tests where the outcome was the greatest mean force output of maximum volunteered contraction, but core endurance expressed by the time of holding the same position during maximum isometric contraction did not correlate significantly with CODS. No significant correlation was found between CODS and core neuromuscular control.

An overview of the studies that investigated the relationship between core strength and CODS is presented in Table 1.

**TABLE 1:** Studies investigating correlations between core strength and CODS in athletes.

Authors	Participants	Core strength test	CODS test	Result
Ahmed et al. (2022)	Male professional badminton players (21.19 ± 1.95 years, n = 36)	Core stability–the pressure of activating core muscles during lower limb movement in mmHg)	Agility *t*-test	Core stability and Agility *t*-test (r = −0.579, *p* < 0.001)
de Bruin et al. (2021)	Female university athletes (n = 83)	Core strength - Biering-Sørensen test–IBE, LF, AF- explosive power output of maximum volunteered contraction in Newtons	Agility *t*-test	Core strength and Agility *t*-test
		Core endurance - Biering-Sørensen test–IBE, LF, AF–duration in seconds		IBE (r = −0.44, *p* < 0.01)
		Core neuromuscular control (NMC)–the changes in pressure of activating core muscles during low-load limb movement		LF (r = −0.39, *p* < 0.01)
				AF (r = −0.44, *p* < 0.01
				Core endurance and Agility *t*-test
				IBE (r = 0.10, *p* = 0.37)
				LF (r = −0.06, *p* = 0.59)
				AF (r = −0.18, *p* = 0.10)
				NMC and Agility *t*-test (r = −0.12, *p* = 0.27)
Cengizhan et al. (2019)	Male professional basketball players (17.0 ± 0.63 years, n = 21)	Core endurance–McGill tests (trunk flexor test, trunk extensor test, side bridge test in seconds)	Hexagonal Obstacle Test (HOT)	Core endurance and HOT
				Trunk flexor (r = 0.288, *p* = 0.320)
				Trunk extensor (r = −0.166, *p* = 0.472)
				Side bridge (r = −0.265, *p* = 0.245)
Imai and Kaneoka (2016)	High school soccer players (16.3 ± 0.5 years, n = 55)	Core endurance–prone and side plunk tests in seconds	The Step 50 agility test	Core endurance and the Step 50 Agility test
				Prone plank (r = −0.436, *p* < 0.01)
				Right side plank (r = −0.222, n.s.)
				Left side plank (r = −0.088, n.s.)
				Combined score (r = −0.365, *p* < 001)
Nesser et al. (2008)	Male National Collegiate Athletic Association Division I football players (n = 29)	Core endurance tests–McGill (trunk flexor test, trunk extensor test, left and right lateral musculature test in seconds)	Pro-Agility Shuttle Run	Core endurance and Pro-Agility Shuttle Run
				Trunk flexion (r = −0.443, *p* < 0.05)
				Trunk extensor (r = −0.356, n.s.)
				Right flexion (r = −0.354, n.s.)
				Left flexion (r = −0.374, *p* < 0.05)
				Total score (r = −0.551, *p* < 0.01)

IBE, isometric back extension; LF, lateral flexion; AF, abdominal flexion.

## 3 Effect of core strength training on CODS

The search strategy included terms such as “effect” AND “core training” AND “change of direction speed” AND “athletes” as well as combinations of their synonyms. The main inclusion criterion was that the studies were experimental in nature, in which core exercises were the main content of the training programs and were applied to young athletes (up to 30 years old) of different sports at a frequency of at least twice a week. Studies were selected independently of gender and level of performance. The studies published before the year 2008 were excluded.

Theoretically, a strong core would transfer forces from the lower to the upper body with minimal dissipation of energy in the torso (Bompa, 1999; McGill, 2009). If power is generated but not transferred, this has a negative effect on performance (i.e., running, jumping, throwing, *etc.*).

In almost all of the studies examined, core strength training lasting six to 9 weeks (one study 12 weeks) was carried out in addition to the usual soccer training. With the exception of three studies (Imai et al., 2014; Prieske et al., 2016; Brull-Maria and Beltran-Garrido, 2021), all consisted of a control group, which only participated in normal soccer training, and one or two experimental groups. During the core strength training programs, almost similar core exercises and training load (frequency per week, core training load, and type of exercises) were performed. There was one study in which core exercises were performed on stable and unstable surfaces (Prieske et al., 2016).

Different results were observed after the application of core strength training on CODS in athletes of different ages and performance levels. For example, a significant improvement in CODS was observed after the application of core strength exercises in middle- and late-adolescent soccer players (Yapici, 2016; Bayrakdar et al., 2020; Brull-Maria and Beltran-Garrido, 2021; Aslan and Kahraman, 2023). In contrast, stabilization and conventional trunk exercises did not contribute to better CODS after 12 weeks of core strength training in 16-year-old soccer players (Imai et al., 2014). A significant improvement in CODS after the application of core strength exercises was also revealed in young, less-skilled soccer players (Afyon et al., 2017; Atli, 2021), while no improvement was observed in higher-skilled players (Prieske et al., 2016; Sever and Zorba, 2018).

An overview of studies that investigated the effect of core strength training on CODS is presented in Table 2.

**TABLE 2:** Studies investigating the effect of core strength training on CODS in athletes.

Authors	Participants	Duration of core strength program	CODS tests	Frequency of trainings and type of core exercises	Result
Afyon et al. (2017)	Amateur soccer players	8 weeks	Illinois Agility Test	EG: 2 × 30 min/week	Significant improvement in both CODS tests performance in, EG (t = 0.172, *p* < 0.001, and t = 0.136, *p* < 0.001)
	Experimental group (EG) (23.17 ± 1.86 years; n = 20)		T-Drill Agility Test	Exercises: jump squat, alternate leg jump, squat, crunch, lying twist trunk, lunge, side plank, burpee, mountain climber, twist with medicine ball + normal soccer training	
	Control group (CG) (22.03 ± 0.50, n = 20)			CG–normal soccer training	
Aslan and Kahraman (2023)	Young male soccer players	6 weeks	Illinois Agility Test	EG: 3x/week, 30–45s application time vs. 45–60s rest, 2 series	Significant improvement in CODS in, EG (t = 8.520, *p* < 0.001)
	Experimental group (EG) - 12.16 ± 0.83 years, n = 12			Exercises: prone plank, side bridge, scissor flutter kick, sit up, superman, bird dog, bicycle crunch, leg lower + soccer team training	
	Control group (CG) (12.25 ± 0.62 years, n = 12)			CG: soccer team training	
Atli (2021)	18–24 years old amateur football players	6 weeks	Illinois Agility Test	EG: 3 × 15 min/week; Exercises: 10 - one side plank, elbow plank, hip extension, mountain climbers, scissors kicks, *etc.*) in 2 series with 20–35s duration + ongoing football training	Significant improvement in CODS in, EG (t = −3.86, *p* = 0.01)
	Experimental (EG) and control group (CG) (n = 20 in each group)			CG: ongoing football training	
Bayrakdar et al. (2020)	12–14-years football players	9 weeks	505 Agility	SEG + DEG: 2x/week; 6 exercises	Significant improvement in both CODS testsperformance in both experimental groups (SEG by 1.05% and 0.46%, respectively, *p* < 0.05, DEG by 2.84%, and 1.62%, respectively, *p* < 0.05)
	Static (SEG) and Dynamic (DEG) exercise group and control group (CG)		Test	SEG: side plank, shoulder bridge, plank, static crunch, leg lift, back extension + normal football training	
	(n = 10 in each group)		Arrowhead	DEG: balance ball with pocket knife, reverse crunch, russian return, shuttle, leg	
			Agility Test	lift, back extension + normal football training	
				CG: normal football training	
Brull-Maria and Beltran-Garrido. (2021)	16–18 years old soccer players	8 weeks	V-cut	SCG + GCS: 2 × 20 min/week; 10 rep. of a 10-s duration with interval of rest 10s	Significant improvement in CODS in both experimental groups (*d* = 1.24, *p* < 0.001, 3.80%).No superiority of any type of core training
	Specific core stability group (SCS) and general core stability group (GCS)			SCC: unilateral skater squat with elastic band, unilateral linear sprint with elastic band, turn and 90° pivot shift with elastic band, lateral lunge with elastic band and their progressions + usual soccer training	
	(n = 7 in each group)			GCG: frontal bridge, dorsal bridge, bird-dog, lateral bridge and their progressions + usual soccer training	
Imai et al. (2014)	16 years old soccer players	12 weeks	Step 50	SE: 3x/week; 40–60s, 2–4 sets	Nonsignificant improvements in CODS in both groups (SE–*p* = 0.212, −1.3%, ES = 0.55, CE–*p* = 0.309, −0.9%, ES = 0.38)
	Stabilization exercises (SE) (n = 10), conventional trunk exercises (CE) (n = 9)			SE–planks, bridges, quadrupet exercises	
				CE–sit-ups, back extension	
Prieske et al. (2016)	Elite soccer players (n = 39)	9 weeks	T - Agility Test	CSTS + CSTU: 2–3x30 min s/week	Nonsignificant improvement in CODS in both groups (CSTS - −0.2%, CSTU–0.6%)
	Core-strengthening exercises under stable (CSTS) and unstable surface (CSTU)			5 exercises were changed every 2 weeks; 15–20s (isometric conditions) or 15–20 rep. (dynamic condition) in 2–3 sets; exercises: prone plank, shoulder bridge, crunches, back extension, side bridges*etc.*	
				CSTS–all exercises on stable surface (floor, bench)	
				CSTU–all exercises on unstable surfaces + regular in-season soccer strength training in both groups	
Sever and Zorba (2018)	Semiprofessional soccer players	8 weeks	505 Agility Test	SG + DG: 3 × 30 min s/week; 25–60s in 2 series; 25–45 rep. in 2 sets	Nonsignificant improvements in CODS in both experimental groups (SG - −1.11%, and −0.7%, respectively, DG - −1.70%, and −0.73%, respectively)
	Static group (SG) - 18.2 ± 1.8 years (n = 14)		Arrowhead Agility Test	SG–side plank, shoulder bridge, crunch, leg raise, back extension + normal soccer training	
	Dynamic group (DG) −17.3 ± 0.6 years (n = 13)			DG–swiss ball jackknife, reverse crunch, Russian twist, leg raise, back extension + normal soccer training	
	Control group (CG) - 17.7 ± 1.3 years (n = 11)			CG–normal soccer training	
Yapici (2016)	Amateur soccer players	6 weeks	Zig-Zag Agility Test	EG: 3x/week; 15 s with rest interval of 60s in 2–3 sets	Significant improvement in CODS in both groups (EG–Z = −2.38, *p* = 0.02, CG–Z = −2.52, *p* = 0.01)
	Experimental group (EG) - 13.75 ± 0.46 years; (n = 16)			Exercises: plank, side plank, crunch, bird dog, shoulder bridge, ball abductor crunch, squat, lunge + football training	
	Control group (CG) - 13.71 ± 0.34 years (n = 16)			CG–football training	

## 4 Discussion

In most cross-sectional studies, a significant association between core strength and COD performance was observed (Nesser et al., 2008; Imai and Kaneoka, 2016; de Bruin et al., 2021; Ahmed et al., 2022). However, the results indicate that this association is highly influenced by the core strength parameter, which is related to CODS. For example, the findings by de Bruin et al. (2021) revealed significant correlations between CODS and the greatest mean force output of the maximum volunteered contraction in the McGill´s core strength tests, but not with total time in their endurance versions. These authors acknowledge that athletic performance in different sports is associated with different components of core stability. Although performance in terms of total score in isometric core endurance tests (planks) was significantly correlated with CODS (Nesser et al., 2008; Imai and Kaneoka, 2016), nonsignificant correlation was revealed with the time in lateral planks (Nesser et al., 2008; Imai and Kaneoka, 2016). Cengizhan et al. (2019) did not confirm the relationship between CODS and endurance of core muscles when the plank tests were used. On the other hand, CODS was associated with core strength when the parameter was the pressure gauge during lower limb movement (Ahmed et al., 2022) and core strength was measured by the maximum volunteered contraction in plank tests (de Bruin et al., 2021). It seems that the assessment of core strength should not include isometric core endurance tests such as planks, back extensions, *etc.*, which do not reflect the dynamic movements specific to many sports. Core strength endurance tests require subjects to maintain a static muscle contraction over a prolonged period of time in trunk flexion, extension and lateral flexion. Athletic performance is primarily dynamic and intermittent, whereas static tests are performed in a non-functional static position, and they do not reflect the actual demands of sport-related activities (Shinkle et al., 2012). It seems that gender, age, and level of performance has not a significant effect on the relationship between core strength and CODS.

Based on the results of cross-sectional studies, this narrative review also analysed studies that investigated the effect of core training on the CODS. The core muscles provide the body’s stability in connection with the skeletal system of the trunk area and has a positive effect on athletic performance (Behm et al., 2010). It plays a pivotal role in effective biomechanical function to generate force and reduce joint loading (Kibler et al., 2006). Six out of nine experimental studies confirmed the positive effect of core strength training on CODS (Yapici, 2016; Afyon et al., 2017; Bayrakdar et al., 2020; Atli, 2021; Brull-Maria and Beltran-Garrido, 2021; Aslan and Kahraman, 2023), while three of them did not observe any significant improvement in CODS after core strength training (Imai et al., 2014; Prieske et al., 2016; Sever and Zorba, 2018). The results suggest that core strength training improves COD performance mainly in middle and late-adolescent athletes (Yapici, 2016; Bayrakdar et al., 2020; Brull-Maria and Beltran-Garrido, 2021; Aslan and Kahraman, 2023) or less-skilled athletes (Yapici, 2016; Afyon et al., 2017), while the effect was not significant in semiprofessional and elite athletes (Prieske et al., 2016; Sever and Zorba, 2018). This can be attributed to their higher CODS level, so that the additional core training does not provide sufficient stimulus for its improvement. There was only one study that did not meet these criteria (Imai et al., 2014), which may be attributed to the absence of a control group and the more coordinatively demanding CODS test. It is more likely that other factors played a role in the Step 50 test than in the usual, less coordination-demanding CODS tests.

To sum up, it seems that core muscle strength plays an important role in the change of direction speed. However, the ability to generate the highest possible activation force of the core muscles in a short period of time is more important for an effective speed of change of direction rather than the endurance strength of core muscles. Core strength training is effective for improving CODS when applied to adolescent or less-skilled athletes. Core programs of semi-professional and professional athletes’ should include more functional and sport-specific core exercises with closed kinematic chains to improve CODS.

There is a need to provide further insight into the relationship between core strength and CODS identify the most appropriate core test variables that more closely reflect athletic performance. The correlation between maximal core strength and performance in CODS tests is also necessary to determine to what extent the core muscle strength contributes to better CODS.
